# Selective attention for affiliative and agonistic interactions of dominants and close affiliates in macaques

**DOI:** 10.1038/s41598-020-62772-8

**Published:** 2020-04-06

**Authors:** Oliver Schülke, Natalie Dumdey, Julia Ostner

**Affiliations:** 1 Department Behavioral Ecology, JFB Institute for Zoology and Anthropology, Georg-August University Göttingen, Kellnerweg 6, 37077 Goettingen, Germany; 2 Research Primate Social Evolution, German Primate Center, Goettingen, Germany; 3 Leibniz ScienceCampus Primate Cognition, Goettingen, Germany

**Keywords:** Social evolution, Animal behaviour

## Abstract

Monitoring conspecifics is a crucial process in social learning and a building block of social cognition. Selective attention to social stimuli results from interactions of subject and stimulus characteristics with dominance rank often emerging as an important predictor. We extend previous research by providing as stimuli naturally occurring affiliative interactions between group members instead of pictorial or auditory representations of conflicts, and by extending to the affiliative relationship, i.e. social bond, between subject and stimulus instead of just their dominance relations. Our observational data on adult female rhesus macaques support the prediction that subjects pay more attention to affiliative interactions of others than to solitary controls. Exceedingly more attention was paid to conflicts unfolding in the group which can have more prompt and direct consequences than others’ friendly interactions. The valence of the stimulus (affiliative vs. agonistic) affected biases towards individuals dominant over the subject, but not the ubiquitous bias towards close affiliates of the subject. Keeping track of the whereabouts and interactions of key social partners has been proposed as a prerequisite for behavioral coordination among bonded partners. In groups of socially very active monkeys, social attention is gated by both social dominance and social bonding.

## Introduction

Vertebrates living in social groups evolved a suite of socio-cognitive skills to maneuver their social environment^1^. These skills include individual recognition, tracking one’s own past interactions, monitoring others’ interactions, assessing others’ relationships, and attributing mental states to others. Such abilities are thought to place increasingly large demands on the cognitive apparatus^1^. Processes involved in the use of social knowledge are a) the acquisition of social information, i.e. information on other individuals and their interactions, b) the application of such information, and c) its exploitation^2^. Each of these processes is subject to phenotypic constraints leading to inter-individual variation in the use of social knowledge^2^. There exists now ample evidence both for the ability to individually recognize conspecifics^3^ and for gregarious animals to take into account past events when making social decisions^4^. Therefore, the focus of social cognition research shifted to understand variation in social attention, in social information acquisition, and in how and when individuals monitor others.

Social information can function as a reward and incentive for nonhuman primates in experimental tasks suggesting that the acquisition of social information is important^5–7^. Not all information is valued to the same degree however, leading to selective attention varying with traits of the subject, of the stimulus individual or event, or their combination. Social information is thought to be valued particularly high, if its acquisition leads to direct benefits for the attentive subject. Selective attention to less immediate, indirect or slowly accruing benefits has been investigated to a lesser degree.

Research on within group variation in social attention established that social attention is gated by dominance rank in primates, leading to an “attentional structure” reflecting the dominance hierarchy in a social group^8–10^. This pattern of selective attention towards higher ranking individuals^9^ along with competition for attractive high ranking partners^11^ has since been found in a number of species and using different paradigms from natural observations to experimental designs^7,12–14^; a recent study lacked a dominance effect though^15^. In eye tracking experiments, the rank-class of the stimulus individual determined whether monkeys had to be provided with rewards to maintain attention and whether subjects visually followed the stimulus with high-ranking individuals receiving more attention even if it had to be paid for^7,16^. Social attention also varies with subject rank; only high-ranking subjects showed late reactions suggestive of voluntary attention sensitive to stimulus rank^16^; low ranking subjects showed a fast, quasi-reflexive reaction that was not modulated by stimulus dominance rank. Subject rank also affects the relative attention focused on a dominant stimulus versus a stimulus encoding information about a potential mating partner^7^. Thus, individual characteristics may interact with traits of the stimulus individual (dominance and sexual attractiveness). It is less clear whether the affiliative relationship between the subject and the stimulus affects social attention.

Apart from paying attention preferentially to high-ranking group mates, individuals may preferentially attend to close affiliates or kin, yet studies on this topic are relatively scarce. Acoustic playback experiments suggest that primates attend more to fear screams^17^ and alarm calls^18^ from closely affiliated, i.e. bonded compared to non-bonded stimulus providers. In an observational study, female mandrills glanced more often at their female kin than at non-kin, and generally attended more to conflicts compared to neutral situations^13^. Social information on the agonistic interactions of one’s close affiliates gains salience, because aggression often is redirected to affiliates of the original opponents^19^. Bonded partners are also recruited preferentially for agonistic support to join in the ongoing conflict^20^. Therefore, it pays to monitor the behavior of one’s affiliates to be prepared. The agonistic interactions of one’s close affiliates may directly and promptly affect the subject.

The affiliative interactions of group members are less likely to have direct and immediate effects on a subject, which may partly explain the pronounced imbalance towards aggression and dominance differentials as stimuli in attention studies (see references above). If a close affiliate is involved in a friendly interaction, salience may accrue from the social information not only about the specific interaction, but also about the dyadic affiliative relationship of or immediate exchanges among the observed individuals. Close affiliative relationships, which are similar to human friendships^21,22^, are formed and maintained by affiliative interactions and often serve as stable alliances in within-group conflicts^23–25^. Thus, monitoring others’ affiliative interactions may serve to keep track of others’ social relationships and indirectly the state of the alliance network in the group, as has been shown for ravens^26^. Beyond information about the alliance network, monitoring others also provides information about the current value of commodities exchanged among partners and relative partner value^27–29^.

The studies reviewed above suggest that any selectivity in social monitoring is driven by the salience of the information gathered (social salience hypothesis). Alternatively, social attention may be focused on close affiliates irrespective of their current activity, the type of social interaction they are involved in or their interaction partners^30^. The frequent joint activities that characterize social bonds need partners to coordinate behaviorally^31^ which requires both or at least one partner to modify its behavior to match the partner’s activity. Such adjustment requires individuals to pay attention to their close affiliates’ whereabouts and behavior at all times^30,32^ (social bonding hypothesis).

Here, we assessed in female rhesus macaques (*Macaca mulatta*) a subject’s selective attention to spontaneous naturally occurring affiliative and agonistic interactions among her group members, and to solitary individuals as controls. These stimulus interactions were scored, for each of the bystander subjects, for whether they involved a) at least one of the subject’s two closest affiliates, and b) at least one individual higher ranking in dominance than the subject. As main effects, we predicted i) that subjects pay more attention to social interactions than controls and because of the imminent risk more to agonistic than to affiliative interactions, ii) that subjects pay more attention to social interactions or controls if they involve at least one of their closest affiliates, and iii) if they involve a group mate higher ranking than themselves. From the social salience hypothesis, we further predicted iv) that involvement of higher ranking or closely affiliated stimulus individuals have exceedingly stronger effects when comparing controls to affiliation and on to agonistic stimulus interactions. To scrutinize this prediction a bit more, we predicted v) that more attention should be paid to the interaction of a close affiliate with an individual higher instead of lower ranking than the subject bystander. From the social bonding hypothesis, we predicted that vi) close affiliates should always be monitored irrespective of whether they are alone, in affiliation or in conflict with a third party.

## Methods

### Study subjects and data collection

The study was conducted in the colony of rhesus macaques housed at the German Primate Center Göttingen, Germany. We observed two groups, one comprising eight adult females age 9–10 without males or immatures and one with a subadult male and nine adult females age 3–23 without immatures. The smaller group was housed in a ~24 m² indoor plus ~18 m² outdoor enclosure and the larger group had access to two of these indoor and outdoor enclosures. The monkeys could move freely between indoor and outdoor area and between rooms, apart from brief periods during some of our observations (see below).

To assess the affiliative relationships between all group members, we conducted instantaneous group scan observations^33^ at 10 min intervals using handheld computers (HP ipaq 114). Each subject was scored only once with priority of grooming over contact sitting and contact sitting over being in close proximity of <1 m. If the individual had more than one partner in the highest priority category, only one partner was chosen alternating going with or against the direction of the clock. A total of 615 scans were recorded in the larger and 625 in the smaller group over the course of five weeks.

Additionally, and separate from these group scans, we collected data on social attention to agonistic and friendly interactions or solitary controls in 77 two-hour sessions (38 sessions in the large and 39 in the small group). Attention was scored only for those subjects that were in the same room as the stimulus individuals and had an unobstructed view on the scene. The reaction of all females to every affiliative (body contact, grooming) and agonistic (including one or more of the following behavioral elements: open mouth display, pointing posture, push and pull, head bob, lunge, chase, slap, bite, ground slap, make room, flee, crouch) interaction between two identified group mates was recorded. Upon the start of the social interaction, we recorded the nature and participants of the interaction and only once for each subject female, like in a quick group scan, whether she directed her attention to the interaction or not, judging from head orientation and gaze direction. Therefore, the likelihood of recording a case of visual attention was not related to the duration of the interaction. During our observations we may have missed gazes that occurred shortly before or after the group scan, but we consider it unlikely that such effects may have biased our data in relation to our predictions. The 1,359 stimulus interactions were scored for whether they involved noises (vocalizing, grooming-noises, self-scratching, cage rattling, loud chewing) or movements (any form of locomotion) which might have drawn attention. A total of 1,158 control observations were conducted of attention paid towards solitary stimulus individuals that were noisy or moved. Data were generated by two observers (813 and 1704 observations balanced between groups). Parallel observations were conducted for two sessions (12 h) during which 81 stimulus events and 302 bystander reactions were scored. Observers agreed on 86.4% of the bystander reactions and inter-observer reliability for attention scoring was estimated at Cohen’s Kappa of 0.7 (R package “irr”^34^) indicating substantial agreement^35^.

### Data analysis

Dominance hierarchies were generated from the outcomes of 235 and 355 decided dyadic agonistic conflicts^36^ using normalized David’s Scores^37^ generated with the function “DS” in the R package “EloRating” version 0.43^38^ in R version 3.4.4^39^. In testing our predictions, we used normalized David’s Scores as a continuous measure of dominance.

Affiliative relationship strength was assessed from the instantaneous group scan data. Relative observation frequencies of grooming, contact sitting, and close proximity were combined from both partners into a dyadic value per behavior and scaled by dividing by the group mean. We then built a dyadic composite sociality index DSI^40^ from those affiliative behaviors that correlated in row-wise matrix correlations run on dyadic data in MatMan 1.1 (Noldus, Wageningen, NL). The DSI is one on average and increasingly high values represent dyads spending exceedingly more time affiliating than the average dyad in the group, whereas lower values indicate dyads that were rarely seen affiliating^41^. Close affiliates were classified for each female as the two partners with whom she had the highest DSI values. Average DSI was 2.3 across all close affiliates, meaning they affiliated more than twice as long as the average dyad in the group.

All further analyses were performed in R [version 3.4.4^42^], by using the Rstudio interface [version 1.0.153^43^]. To test what influenced the probability of a bystander to pay attention to a stimulus or not, we ran two Generalized Linear Mixed Models (GLMM) with a binomial error structure and logit link function, using the function ‘glmer’ of the R package lme4^44^. In the first model, we included the two statistical interaction terms i) between the stimulus type (control, affiliation or aggression, also called stimulus valence in the text) and the engagement of a close affiliate of the subject bystander in the stimulus interaction and ii) between the stimulus type and the engagement of a higher ranking individual than the subject bystander in the stimulus interaction, and iii) all three main effects, as well as iv) the rank of the subject bystander as fixed effects. After we found that the first interaction term was not significant (see Table S1), we excluded it and reported in the main text results from the reduced model. For the second model, we used only the subset of the data where only one close affiliate of the subject bystander was present in a social interaction; the full data set also included stimuli where both interaction partners were close affiliates. In this model, we included the interaction between the stimulus type (affiliation or aggression) with whether the member of the social interaction who was not a close affiliate was higher ranking than the subject bystander or not. For both models we also included the conspicuousness of the stimulus (whether it was noisy, also involved movement or none of the two) as a control variable, and included event ID, bystander ID and actor and receiver of the social interaction as random effects. Using a likelihood ratio test, we compared the full model with a null model lacking the fixed effects, but comprising the same control and random effects to test whether the predictors of interest improved model fit. Model stability was assessed by comparing the model estimates of the full dataset with the estimates of the model after leaving out data points one by one. No influential cases were detected. We checked whether predictors were collinear by calculating Variance Inflation Factors (VIF^45^) for a linear model, excluding the random effects. Since VIFs were close to one for all models, collinearity was not an issue.

This work followed the Animal Behaviour Society’s guidelines for the treatment of animals in behavioral research and teaching, and adhered to standards as defined by the European Union Council Directive 2010/63/EU on the protection of animals used for scientific purposes. The study was approved by the Ethics Committee of the German Primate Center Göttingen (AZ E1-19).

All data generated and analyzed during this study are included in this published article and its supplemental information files.

### Ethical approval

All applicable international, national, and/or institutional guidelines for the care and use of animals were followed. The Ethics Committee of the German Primate Center approved of this study (AZ E1-19) that was completely observational.

### Results

**Figure 1:**
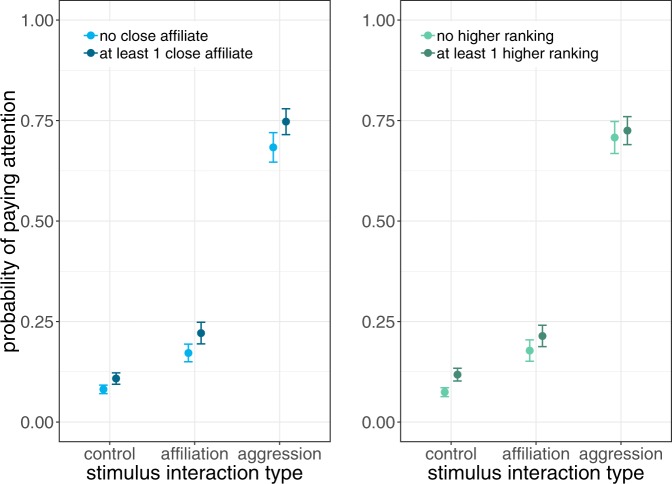
Probability of subjects paying attention to stimuli increased from solitary controls to affiliative interactions and on to agonistic interactions as stimuli. A similar bias towards stimuli that involved at least one of the subject’s two closest affiliates is expressed across stimulus types. Stimuli that involved at least one individual higher ranking than the subject evoked more attention only if they were controls or affiliative interactions but not if agonistic. See Table 1 for detailed results of GLMM.

The affiliative and the dominance relationships between the subject and the stimulus individuals modulated subject attention in different ways. Whether the stimulus situation involved a close affiliate of the subject or not did not modulate the effect of stimulus type on attention (Table S1). Subjects always were more attentive to a close affiliate with the size of this effect being independent of whether the stimulus interaction was agonistic, affiliative or a solitary control (Table 1; Fig. 1a).

**Table 1:** Predictors of rhesus macaque social attention to spontaneously occurring natural social stimuli.

		Estim.	StdErr	z	Pr(>|z|)
**Stimulus type/valence**					
	**Affiliative vs. control**	**0.99**	**0.15**	**6.44**	**<0.0001**
	**Agonistic vs. control**	**3.40**	**0.16**	**20.79**	**<0.0001**
	**Affiliative vs. agonistic**	**2.41**	**0.18**	**13.69**	**<0.0001**
**At least one close affiliate (yes)**		**0.30**	**0.06**	**4.69**	**<0.0001**
**At least one higher ranking (yes)**		**0.55**	**0.14**	**3.97**	**<0.0001**
**Stimuls type:One higher ranking (yes)**					
**One higher ranking (yes)**	**Affiliative vs. control**	**−0.29**	**0.17**	**−1.71**	**0.087**
**One higher ranking (yes)**	**Agonistic vs. control**	**−0.42**	**0.17**	**−2.54**	**0.011**
**One higher ranking (yes)**	Agonistic vs. affiliative	−0.14	0.18	−0.79	0.430
Subject dom. rank (sqrt nDS)		−0.09	0.11	−0.90	0.367

Results of a logistic model (whether subject gazed at stimulus) controlling for conspicuousness of the stimulus (whether it was noisy, involved movement or none of the two) and including as random effects stimulus event ID, subject ID, ID of actor and receiver; 10.162 observations, 2.478 stimulus events, 18 subjects. The full model was significantly different from the null model including all control and random effects (Chi^2^ = 362.05, df = 7, P < 0.0001). Significant predictors are presented in bold font. Significance of the different levels of variable stimulus type was assessed by releveling the intercept.

**Figure 2:**
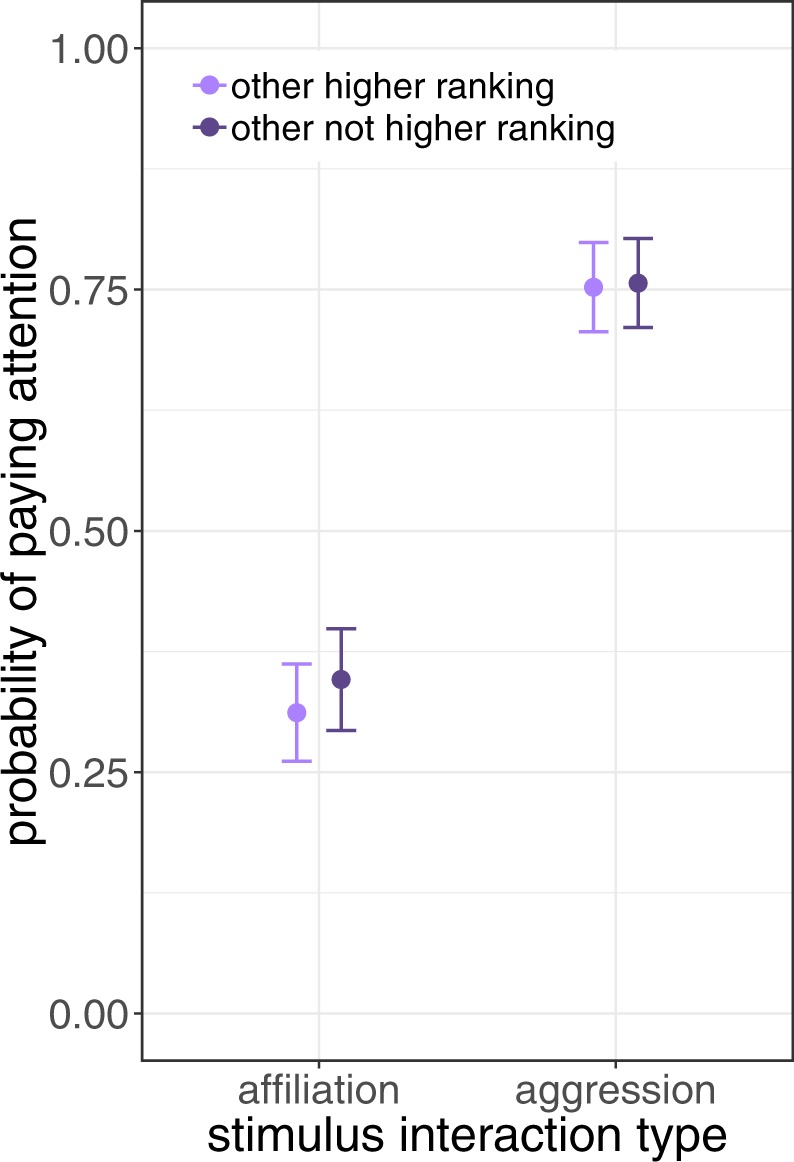
Subset of the data with stimulus interactions that had exactly one of the two closest affiliates of the subject and the other stimulus individual being dominant over the subject or not, to test whether a close affiliate in conflict is more likely to be monitored if the opponent is a bigger threat to the subject. The opponent’s dominance rank relative to the subject did not affect attention. See Table 2 for detailed results of GLMM.

In contrast, the dominance relationship between subject and stimulus individual(s) did not affect subject attention the same way across stimulus types. Involvement of a higher ranking individual only raised more attention for control or affiliative interactions; surprisingly, no such difference was found for agonistic interactions perhaps due to a ceiling effect (Table 1; Fig. 1b). Beyond these relative effects, the dominance rank of the subject had no effect on its attention. In the subset of stimulus events that included only one close affiliate of the subject, the dominance relation between the second - not affiliated - stimulus individual and the subject did not affect social attention (Table 2; Fig. 2). Females did not pay more attention to the interaction of their close affiliates with higher ranking individuals, not even if they were engaged in an agonistic interaction.

**Table 2:** Model results for subset of stimulus interactions that had exactly one close affiliate and the other individual being higher ranking than the subject or not.

	Estim.	StdErr	z	Pr(>|z|)
**Stimulus type (agonistic vs. affiliative)**	**2.27**	**0.22**	**10.12**	**<0.0001**
Other higher ranking (yes)	0.31	0.21	1.51	0.131
Stimulus Type:Other higher ranking	−0.20	0.24	−0.80	0.423
Subject dom. rank (sqrt nDS)	−0.08	0.16	0.52	0.606

The crucial interaction term stimulus type:other higher ranking is not significant. The full model is different from the null model with control (stimulus conspicuousness) and random effects (stimulus event ID, subject ID, actor ID, recipient ID; Chi^2^ = 292.6, df = 5, P < 0.0001; 2169 observations, 1123 stimulus events, 18 subjects).

Overall, the valence of the stimulus/stimulus type was by far the most important predictor of attention. Agonistic interactions raised much more attention than affiliative ones, but both raised more attention than control situations with a solitary stimulus individual. All analyses were controlled for the conspicuousness of the stimulus, i.e. whether it involved noise/vocalization and movement.

## Discussion

Arguably, the most likely direct and prompt/immediate benefit of paying attention to others is the avoidance of danger by monitoring potential threats. To do so, individuals should monitor individuals and situations that may carry the risk of themselves becoming involved in an aggressive interaction. Indeed, mandrills were more attentive to conflict situations compared to neutral ones^13^, rhesus macaques looked more rapidly at pictures of aggressive compared to neutral facial displays of conspecifics^46^, and Barbary macaques glanced longer at anxious (i.e. scratching) weakly bonded than strongly bonded group members, possibly as the former may be more likely to start a fight^47^. Our findings, that third party conflicts drew far more attention from the subject bystander than affiliative or solitary control situations corroborate these results.

An efficient way to avoid threat and resource competition is to generally keep track of the whereabouts of one’s potential opponents regardless of the opponent’s specific behavior. The widespread primate inclination to focus attention on higher-ranking individuals supports this idea^30^. Female rhesus macaques in our study also paid more attention to individuals outranking themselves but only if the higher ranking individual was alone or in an affiliative interaction. Agonistic interactions were almost always monitored no matter whether they involved an opponent higher ranking than the subject or not. Previous studies also found low ranking subjects to be more attentive and possibly more unselectively so^16,47^, whereas high social status may require selective attention only to other high rankers^16^. Yet, in mandrills and the rhesus macaque females in our study such an effect of subject rank on attention for agonism and affiliation of others was not found to go above and beyond the relative effects described above^13^. This discrepancy may be explained by rank dependent differences in the time course of gazing^16^ that cannot be picked up with the methods employed in the latter studies (but see^14^). The methods employed here do not allow discerning which of the interaction partners the subject was gazing at.

Threats can also be avoided by selectively monitoring third party conflicts that involve a close affiliate of the subject. Such monitoring may prepare closely affiliated bystanders for upcoming interactions. First, as it has been shown that redirection of aggression is preferentially directed towards bond partners of the original opponents^19^, closely observing conflicts involving affiliates may increase the chance to avoid becoming target of redirection. Secondly, selective attention towards close affiliates’ aggressive interactions may also enhance behavioral coordination needed to join in the ongoing fight in case the affiliate calls for coalitionary aid and agonistic support^20,30^. Thus, there are several direct benefits of monitoring conflicts of group members, which may be amplified if one of the opponents is a bonded partner. Accordingly, subjects in this study were sensitive to whether a nearby conflict involved one of their close affiliates, but the bias towards monitoring close affiliates in agonistic interaction did not exceed the bias towards close affiliates in affiliative or control situations. Subjects did not pay more attention if the opponent in a conflict of their affiliate was higher ranking than themselves and therefore posed a bigger threat, which may result from a ceiling effect of very high attention to agonistic interactions.

A different useful yet more indirect source of information that is rarely investigated in studies of social attention are affiliative interactions of third parties. Monitoring friendly interactions of group members provides the observer with valuable information on others’ social relationships^1,30,31^ as well as the value of commodities exchanged among group members^48,49^. Keeping track of the status quo and dynamics of social ties in one’s group aids in social decision making, for example in strategic partner choice for coalition formation or in the decision of whether or not to attack a specific target^50,51^. Given the importance of bonds and alliances in some social species^52–54^, it should pay to closely track others’ affiliative interactions to possibly impede emerging bonds^26,55,56^. Sooty mangabeys, chimpanzees, and horses strategically intervene in grooming interactions threatening their own relationship status and those that involved a close affiliate of the bystander^55,56^. Female rhesus macaques in this study paid more attention to third party affiliation if it involved an individual higher ranking than themselves possibly because social exchanges and relationships of powerful individuals have more far reaching consequences. The subjects further exhibited an attentional bias towards the affiliative interactions of their own close affiliates, but this bias did not exceed the bias towards monitoring solitary close affiliates.

Differences in attention towards close affiliates and others were not modulated by stimulus valence but were equally strong for agonistic, affiliative, and solitary stimulus events. The bias towards close affiliates was not exacerbated by increasing the directness and immediacy of the threat involved. Instead, our results can be interpreted as a general attention bias towards close affiliates independent of the affiliate’s social activity. Such ubiquitous close monitoring of affiliates has been proposed as a prerequisite for behavioral coordination and adjustment of own behavior to achieve this goal and therefore may serve as an index of social bond strength^30^. Investing in such close monitoring may have opportunity costs in terms of reduced feeding efficiency or reduced attention to other, non-bonded individuals. Like any visual monitoring it may also attract the attention of those observed with possible negative effects like redirected aggression. The upside is that social monitoring may also facilitate social learning which may be particularly important for immatures^14^. Future studies will explore the immediate consequences of social monitoring.

In conclusion, this study partly replicates previous findings on selective attention to social stimuli in nonhuman primates, particularly the general attraction to dominant individuals. We extend previous work in several ways. We show selective social monitoring of a dominant’s affiliative interactions that should not have direct and prompt effects on the subject’s wellbeing like extending agonistic conflicts do. We show that biases towards monitoring higher ranking individuals disappear when monitoring agonistic interactions even if these involve close affiliates. We further show that attention to the whereabouts and interactions of one’s own close affiliates is generalized which suggests that it serves in coordinating the affiliative relationship itself. As a side effect, the individual gains more social information about its closest partners than other group members. Social interest is typically assessed with pictures^7^, acoustic playbacks^1^ and videos^46^ as stimuli or uses gaze following paradigms with human observers^57^. Here, we recorded the reaction of bystanders to spontaneously occurring social interactions among other group members. The design allowed statistically disentangling the effects of stimulus valence (agonistic, affiliative and non-social) as well as affiliative and dominance relationships between stimuli and subjects on attention. If applied in field studies the design will allow to assess the consequences of inter-individual differences in social monitoring in terms of efficacy of coalition partner choice and longer-term fitness outcomes.

## Supplementary information

Supplementary Results.

Supplementary Dataset 1.

Supplementary Dataset 2.

## Data Availability

All data generated and analyzed during this study are included in this published article and its supplemental information files.
