# Supplementary material for: Selective attention for affiliative and agonistic interactions of dominants and close affiliates in macaques
Source: Sci Rep. 2020 Apr 6;10:5962. doi: 10.1038/s41598-020-62772-8 (PMC7136223; doi:10.1038/s41598-020-62772-8)
Supplement: Supplementary file 1 — Supplementary Results. [file 41598_2020_62772_MOESM1_ESM.docx]

# Supplementary Material for

Selective attention for affiliative and agonistic interactions of dominants and close affiliates in macaques

Oliver Schülke^1,2,3*^ & Natalie Dumdey^1,3^, Julia Ostner^1,2,3^

Table S1: Full model with both interaction terms is significantly different from null model with controls and random factors (Chi²=364.4, df=9, P<0.0001). The Interaction term Stimulus type:One close affiliate is not significant (LRT=2.33, P=0.312). A reduced model without the interaction term is presented in the manuscript. Reported here are results of a logistic model (whether subject gazed at stimulus or not) controlling for conspicuousness of the stimulus (whether it was noisy, also involved movement or none of the two) and including as random effects stimulus event ID, subject ID, ID of actor and receiver; 10162 observations, 2478 stimulus events, 18 subjects. Significance of the different levels of variable stimulus type was assessed by reveling the intercept.

|  |  | Estim. | StdErr | z | Pr(>\|z\|) | Pr(Chi) |
| --- | --- | --- | --- | --- | --- | --- |
| **Stimulus type** |  |  |  |  |  |  |
|  | **Affiliative vs. control** | **0.91** | **0.17** | **5.50** | **<0.0001** |  |
|  | **Agonistic vs. control** | **3.35** | **0.17** | **19.30** | **<0.0001** |  |
|  | **Agonistic vs. affiliative** | **2.44** | **0.19** | **12.78** | **<0.0001** |  |
| One close affiliate (yes) |  | 0.18 | 0.10 | 1.70 | 0.089 |  |
| **One higher ranking (yes)** |  | **0.54** | **0.14** | **3.86** | **0.0001** |  |
| Stimulus type:One close affiliate (yes) |  |  |  |  |  | 0.312 |
| One close affiliate | Affiliative vs. control | 0.21 | 0.15 | 1.42 | 0.16 |  |
| One close affiliate | Agonistic vs. control | 0.18 | 0.16 | 1.14 | 0.25 |  |
| One close affiliate | Agonistic vs. affiliative | -0.18 | 0.18 | -0.78 | 0.435 |  |
| **Stimuls type:One higher ranking (yes)** |  |  |  |  |  | **0.031** |
| One higher ranking | Affiliative vs. control | -0.28 | 0.17 | -1.70 | 0.088 |  |
| **One higher ranking** | **Agonistic vs. control** | **-0.43** | **0.17** | **-2.53** | **0.012** |  |
| One higher ranking | Agonistic vs. affiliative | -0.14 | 0.18 | -0.78 | 0.435 |  |
| Subject dom. rank (sqrt nDS) |  | -0.10 | 0.10 | -0.95 | 0.344 |  |

The full data set and the reduced data set of stimulus events with ony exactly one close affiliate oft he bystander are provided is separate csv file.
